# The role of probiotics in vannamei shrimp aquaculture performance – A review

**DOI:** 10.14202/vetworld.2023.638-649

**Published:** 2023-03-27

**Authors:** Muhammad Kholiqul Amiin, Almira Fardani Lahay, Rizha Bery Putriani, Muhammad Reza, Septi Malidda Eka Putri, Md. Afsar Ahmed Sumon, Mamdoh T. Jamal, Muhammad Browijoyo Santanumurti

**Affiliations:** 1 Department of Marine Science, Faculty of Agriculture, Universitas Lampung, Bandar Lampung, Indonesia; 2 Department of Aquatic Resources, Faculty of Agriculture, Universitas Lampung, Bandar Lampung, Indonesia; 3 Department of Aquaculture, Faculty of Agriculture, Universitas Lampung, Bandar Lampung, Indonesia; 4 Department of Marine Biology, Faculty of Marine Sciences, King Abdulaziz University, Jeddah, Kingdom of Saudi Arabia; 5 Department of Aquaculture, Faculty of Fisheries and Marine, Universitas Airlangga, Surabaya, Indonesia

**Keywords:** application, bacteria, farm, microbiome, shrimp

## Abstract

Vannamei shrimp (*Litopenaeus vannamei*) is an important food commodity of economic benefit due to its high price, low susceptibility to disease, and popularity for consumption. These advantages have led many farmers to cultivate vannamei shrimp. Efforts are underway to improve the aquaculture performance of this species, including the use of probiotics, which are non-pathogenic bacteria that aid in digestion and help fight disease. Probiotics are usually obtained from the intestines of vannamei shrimp or the culture environment. They are low-cost, non-pathogenic, and largely non-toxic source of antibiotics and are able to synthesize various metabolites that have antibacterial functions and applications. Research on probiotic use has primarily been focused on increasing vannamei shrimp aquaculture production. Bacterial species, such as *Lactobacillus* or *Nitrobacter*, can be administered orally, by injection, or as a supplement in aquaculture water. Probiotics help to improve survival rate, water quality, immunity, and disease resistance through space competition with disease-causing bacteria, such as *Vibrio* spp. An increased number of probiotic bacteria suppresses the growth and presence of pathogenic bacteria, which lowers disease susceptibility. In addition, probiotic bacteria also aid digestion by breaking down complex compounds into simpler substances that the body can absorb more easily. This mechanism improves growth performance in terms of weight, length, and feed conversion ratio. This review aimed to provide information regarding contribution of probiotic to improve vannamei shrimp production in aquaculture.

## Introduction

Shrimp is an aquaculture commodity with great prospects. Data from global shrimp production report that in 2019, more than 4,100,000 shrimp were produced to meet world demand [1]. As a commodity, shrimp have the largest demand in the world, with a value of 4.85 billion USD [2]. The largest market demand for shrimp is found in the United States of America (40%), Southeast Asia (28%), the European Union (13%), and Japan (6%) [2]. Vannamei shrimp (*Litopenaeus vannamei*) are a popular shrimp species due to their good taste, high nutritional value, well-understood aquaculture methods, and high resistance to disease [3–5]. The majority of vannamei shrimp are produced globally through aquaculture activities (83%) [6].

Many innovations have been developed to improve vannamei shrimp cultures. Probiotics, for example, are non-pathogenic bacteria that live in the host intestine and provide positive health effects [7] by improving the host’s immune system to fight disease and aiding in host development [8]. These beneficial bacteria have been widely used in the shrimp industry and are often supplemented through food and injection [9]. Meta-analysis data from 100 studies have shown that probiotics can increase the survival rate of vannamei shrimp by up to 95% compared to controls [10]. In vannamei shrimp, probiotics strengthen the immune system against pathogenic bacteria, viruses, and environmental factors [11].

This review aimed to provide information regarding the role of probiotics in vannamei shrimp aquaculture performance. The performance parameters reported in this review include growth performance, survival rate, water quality, immunity, and disease resistance. We also define probiotics and discuss their roles in shrimp aquaculture.

## Probiotic Mechanisms to Improve Vannamei Shrimp Aquaculture Performance

The previous studies have shown that bacteria, such as *Bacillus*, *Lactobacillus*, *Enterococcus*, *Alteromonas*, and *Arthrobacter* spp., can improve the performance parameters in shrimp aquaculture (e.g., growth performance, survival rate, immunity, disease resistance, and water quality) [12–17] through multiple mechanisms, including gut colonization, antagonistic activities, digestive enzyme secretion, organic waste removal, and the production of supplemental nutrients (e.g., biotin, Vitamin B12, fatty acids, essential amino acids, and other necessary growth factors) [10]. Before activating these mechanisms, bacteria enter the intestine, restore the composition of the gut microbiome, and introduce functions that are beneficial to the gut microbial community. These activities each improve or prevent intestinal inflammation and other intestinal and systemic disease phenotypes [18]. The intestines provide a comfortable environment for microbiota to grow due to the abundance of nutrients already present in the intestinal ecosystem for the host which is also needed by bacteria [19]. The symbiotic relationships found in the intestines can include mutualism, commensalism, and parasitism, depending on the dominant type of bacteria present [20]. For example, when beneficial bacteria are dominant, shrimp aquaculture performance improves, but if harmful bacteria are dominant, shrimp will be more susceptible to disease, grow more slowly, and even perish. Thus, probiotics function to colonize the gut in beneficial ways.

After entering the host body, probiotics activate insulin-like growth factor 1 (IGF-1) by increasing short-chain fatty acids (SCFA) [21]. Insulin-like growth factor 1 is mainly secreted by the liver as a result of stimulation by growth hormone (GH) [22]. It binds to receptors on the cell surface, and primarily works to activate cell proliferation and differentiation [23]. Short-chain fatty acids also performs antagonistic activities that produce metabolites, such as organic acids, hydrogen peroxide, ethanol, acetaldehyde, acetoin, carbon dioxide, reuterin, and other bacteriocins and play a role in competitive exclusion, immune modulation, the stimulation of host defenses, and the production of signaling molecules that trigger gene expression changes [24]. For example, antagonist activities are involved in histone deacetylase, which causes DNA to become more tightly wrapped around histone cores, thus making it harder for transcription factors to bind to DNA by deacetylating the histone tails [25]. This leads to decreased levels of gene expression and is known as gene silencing [26]. Probiotics deactivate histone deacetylase and elevate GH through gene expression.

Probiotics also help break down nutrients or compounds into simpler forms that are more easily absorbed. According to previous research, probiotics increase digestive enzyme secretion (amylase, protease, and lipase) and produce nutrients (vitamins, fatty acids, and amino acids) that can contribute to the digestive process and feed utilization [27]. The results of these processes are then utilized by the host body for growth, improved survival, and health.

**Figure-1:**
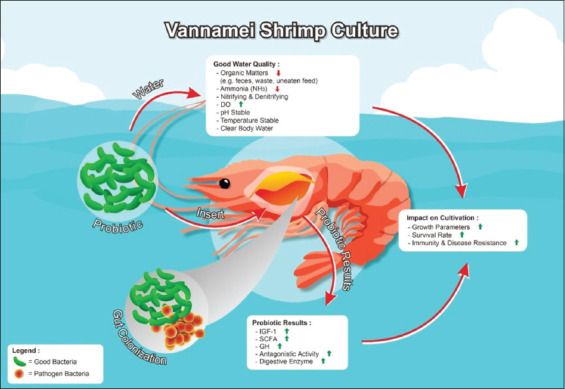
Role of probiotics in vannamei shrimp aquaculture [Source: Figure prepared by the authors].

Probiotics also play an external role (i.e., water quality), in addition to the internal roles that they play. For example, probiotics help decrease the amount of organic material in water caused by, for example, feces, waste, deceased organisms, and uneaten pellets in aquaculture water [28]. This is especially important since the organic matter in the water can turn into ammonia, a highly toxic compound [29] that can cause a mortality rate of up to 100% in vannamei shrimp cultures [30]. Probiotics reduce the ammonia content of water through nitrification and denitrification [31]. In addition, the dissolved oxygen content in the water increases when probiotics are present since they break down organic matter that can then be used by oxygen producers as nutrients to facilitate photosynthesis [32]. The mechanisms involved in the use of probiotics in vannamei shrimp aquaculture are presented in Figure-1.

## Field Applications

**Figure-2:**
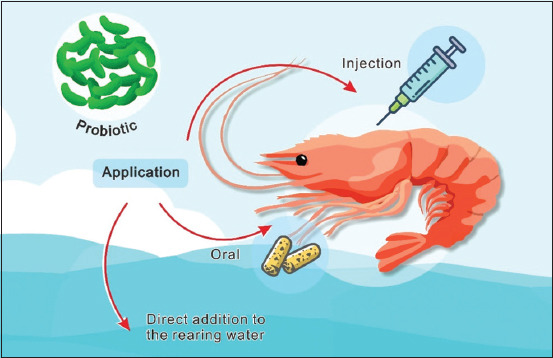
Administration way to deliver probiotics to vannamei shrimp [Source: Figure prepared by the authors].

Probiotics can be classified into several types depending on their application in the field (i.e., in a medium (water) or immersion, oral, and injection) [13, 33, 34]. The administration of probiotics to vannamei shrimp cultures is shown in Figure-2. Probiotics that are added to the water can grow in the water medium by absorbing all the nutrients present in the water [35]. This allows all digestible food present in the water to be absorbed, which results in the starvation of any pathogenic bacteria present due to malnutrition [36]. A drawback of this administration method is that it cannot guarantee that vannamei shrimp present in the water will absorb and use the probiotics (unspecific target) [37].

Oral probiotics are provided to vannamei shrimp in artificial feed to increase the beneficial microflora in the gut [13] and can also be administered through probiotic-rich *Artemia* or microalgae species to improve growth and survival during the feeding phase [38]. Microencapsulation is another method of probiotic administration that directly and positively impacts water quality, physical parameters, and shrimp health [39]. An advantage of this method is that the shrimp obtain a variety of probiotics needed by the body, but this method requires that the viability of the probiotics be checked continuously to ensure that the probiotics are working [39]. Finally, probiotics can be administered directly to the shrimp body as an injection, which ensures that the probiotics have entered the body [40]. The time and cost required to inject each individual shrimp are disadvantages of this method.

## Stimulation of Growth Performance and Survival Rate

Growth performance is an important parameter of vannamei shrimp culture due to the cost and time involved in growing shrimp from a small size to a consumption size [10–12, 38]. Growth performance parameters include specific growth rate, final body weight, weight gain, feed efficiency, and feed conversion ratio (FCR) [41, 42]. These parameters are calculated to determine whether the growth of the vannamei shrimp is optimal, since they affect cultivation success (i.e., cost, profit, and loss) [43]. Several studies related to the role of bacteria in the growth performance of vannamei shrimp are shown in Table-1 [16, 44–55].

**Table-1:** The role of probiotics on growth performance and survival rate in vannamei shrimp culture.

S. No.	Probiotic	Dosage	Administration	Effect	Duration	Density	Reference
1.	*Bacillus coagulans*	1 × 10^6^ (BC1), 1 × 10^7^ (BC2) and 1 × 10^8^ (BC3) CFU/g of feed	Oral with feed	Improving growth (especially 1 × 10^8^ (BC3) CFU/g of feed)	56 days	40	[44]
2.	*C. butyricum*	0, 1 × 10^7^, 1 × 10^8^, 1 × 10^9^, 1 × 10^10^, 1 × 10^11^ and 1 × 10^12^ CFU/kg	Oral with feed	10^11^ and 10^12^ CFU/kg significantly improve the growth performance	42 days	10	[45]
3.	*Lactobacillus plantarum* Ep-M17	5 × 10^8^ CFU g 1	Oral with feed	Increase specific growth rate	4 weeks	50	[46]
4.	*B. subtilis*, lactic acid bacteria	10^5^ CFU (g diet) of *B. subtilis* and 10^5^ CFU (g diet) of LAB, 10^6^ CFU (g diet) of *B. subtilis* and 10^6^ CFU (g diet) of 1 LAB	Oral with feed	Shrimp growth increase, especially 10^5^ CFU (g diet)	12 weeks	200	[48]
5.	*B. subtilis* E20	10^7^ CFU/kg with encapsulated and 10^8^ CFU/kg without encapsulated	Oral with feed	Improve shrimp growth	56 days	60	[49]
6.	*Arthrobacter bussei*	0.2%, 0.4%, 0.6%, 0.8%, and 1.0%	Oral with feed	Significantly higher final body weight, weight gain, feed efficiency, and specific growth than control	6 weeks	25	[16]
7.	*B. subtilis*	Four additional levels of Gutcare^®^ (0, 0.05%, 0.15% and 0.3%) were added under two fishmeal diets (20% and 5% fishmeal levels)	Oral with feed	Regardless of high/low fishmeal diet, it showed higher growth performance compare control (0%)	56 days	30	[50]
8.	*B. subtilis*	0%, 0.2%, 0.5%, 1% of *B. subtilis*, and 0.5% of *B. subtilis* mixed with yeast	Oral with feeding	Increase weight, growth rate, and specific growth rate (especially 0.5% of *B. subtilis* treatment)	30 days	40	[51]
9.	*C. butyricum*	0.03% (C1, 3 × 10^8^ CFU/kg), 0.12% (C2, 1.2 × 10^9^ CFU/kg), 0.48% (C3, 4.8 × 10^9^ CFU/kg) and 1.92% (C4, 1.92 × 10^10^ CFU/kg)	Oral with feed	Increase growth performance (weight gain rate, specific growth rate, survival rate, FCR, survival rate) than control, especially 0.12%–0.48% (1.2 × 10^9^ CFU/kg–4.8 × 10^9^ CFU/kg)	8 weeks	40	[52]
10.	*Psychrobacter* spp.	10^5^ CFU/mL	Immersed to water	Higher weight (40%) and length (5.6%) than that observed in untreated animals	15 days	150	[47]
11.	*Rhodobacter sphaeroides* strains; SS15, S3 W10, TKW17, and *Afifella marina* STW181	1, 3 and 5% (w/w)	Oral with feed	Increase growth rate and survival rate, especially 1% treatment	60 days	600	[53]
12.	*C. butyricum* CBG01	0.1 × 10^7^ CFU/kg 0.1 × 10^8^ CFU/kg 0.1 × 10^9^ CFU/kg 0.1 × 10^10^ CFU/kg 0.1 × 10^11^ CFU/kg 0.1 × 10^12^ CFU/kg	Oral with feed	Shrimp survival rates ranged from 92% to 98%	21 days	10	[54]
13.	*L. pentosus* HC-2 *and Enterococcus faecium* NRW-2	1 × 10^7^ CFU g/feed of *L. pentosus* HC-2, 1 × 10^7^ CFU/g feed *of Enterococcus faecium* NRW-2, and supernatant of strain *L. pentosus*	Oral with feed	97% higher survival rate than without treatment	28 days	100	[55]

LAB=Lactic acid bacteria, *L. pentosus*=*Lactobacillus pentosus*, *B. subtilis*=*Bacillus subtilis*, *C. butyricum*=*Clostridium butyricum*, CFU: Colony-forming unit

As described previously, probiotics can improve shrimp growth by breaking down nutrients or compounds into simpler compounds that are more easily absorbed and by activating GHs [21, 56]. Some of the bacteria reported to help the growth of shrimp are *Bacillus, Clostridium*, *Lactobacillus*, *Psychrobacter*, and *Arthrobacter* spp. [16, 44–46]. These bacteria have been found in the intestines of vannamei shrimp and provide many benefits.

Table-1 also provides information regarding the increased survival rates found in vannamei shrimp with probiotic use. Probiotics help shrimp meet their nutritional needs[57], which helps to protect the organism from stress, malnutrition, and death [11]. The survival rate is an important indicator of successful shrimp cultivation since it directly affects the number of shrimps sold and the farmer’s profits.

## Immune Modulation and Disease Resistance

To increase the shrimp’s resistance to disease infestations, probiotic treatments stimulate non-specific disease resistance in the body. Several diseases found in shrimp cultures are caused by *Vibrio alginolyticus*, *Vibrio parahaemolyticus*, and *Aeromonas, Photobacterium, Tenacibaculum*, and *Shewanella* spp. [58–60]. *Vibrio parahaemolyticus* causes acute hepatopancreatic necrosis disease (AHPND) in vannamei shrimp [61] and is characterized by severe atrophy in the shrimp hepatopancreas that exhibits unique histopathology at the acute stage [62]. This disease, which is also called early mortality syndrome, can cause 40%–100% mortality in shrimp cultures [63, 64]. Table-2 shows how probiotics can induce immune modulation and improve disease resistance, even in the case of AHPND [16, 44, 46–50, 52, 54, 58, 65–70].

**Table-2:** The role of probiotics on immune modulation and disease resistance in vannamei shrimp culture.

S. No.	Probiotic	Dosage	Administration	Effect	Duration	Density per tank	Reference
1.	*L. plantarum*	32.15 mg/L	Oral	Immunity and disease resistance increase	60 days	100	[66]
2.	*L. plantarum* Ep-M17	5 × 10^8^ CFU/g	Oral with feed	Protection immune rate of 76.9%	4 weeks	50	[46]
3.	*B. subtilis* E20	10^7^ CFU/kg with encapsulated and 10^8^ CFU/kg without encapsulated	Oral with feed	Increase immune response and disease resistance of *V. alginolyticus*	56 days	60	[49]
4.	*Arthrobacter bussei*	0.2%, 0.4%, 0.6%, 0.8%, and 1.0%	Oral with feed	Increase innate immunity and antioxidant capacity	6 weeks	25	[16]
5.	*B. subtilis*	Four additional levels of Gutcare^®^ (0, 0.05%, 0.15% and 0.3%) were added under two fishmeal diets (20% and 5% fishmeal levels)	Oral with feed	Regardless of high/low fishmeal diet, it showed higher antioxidant and immune-related enzyme activities compared to the 0% (control)	56 days	30	[50]
6.	*Clostridium butyricum*	0.03% (C1, 3 × 10^8^ CFU/kg), 0.12% (C2, 1.2 × 10^9^ CFU/kg), 0.48% (C3, 4.8 × 10^9^ CFU/kg) and 1.92% (C4, 1.92 × 10^10^ CFU/kg)		Improve antioxidant activity, immunity, and disease resistance than control, especially 0.12%–0.48% (1.2 × 10^9^ CFU/kg-4.8 × 10^9^ CFU/kg)		40	[52]
7.	*Psychrobacter* spp.	105 CFU/mL	Immersed in water	Higher disease resistance of *Aeromonas* spp. than that observed in untreated animals	15 days	150	[47]
8.	*Pseudoalteromonas* CDM8 and CDA22	CDM8 dosed at 10^7^ CFU/kg; CDA22 dosed at 10^7^ CFU/kg; Group C, mixture of CDM8 and CDA22 (at a ratio of 1:1) dosed at 10^7^ CFU/kg	Oral with feed	Increase mortality of higher 96.7% and decrease the presumptive *V. parahaemolyticus* counts	21 days	35	[54]
9.	*Rhodobacter sphaeroides* SS15	0.27% (w/w)	Oral with feed	27% higher than without treatment and no sign of acute hepatopancreatic necrosis disease from *V. parahemolyticus*	63 days	20	[65]
10.	*Lactobacillus* spp.	10^6^ CFU/mL, 10^8^ CFU/mL, and 10^10^ CFU/mL of Lactobacillus spp.	Oral with feed	Increase survival rate value of shrimp by 86.67% from *V. parahemolyticus* infection	30 days	10	[67]
11.	*Lactobacillus acidophilus*	1 × 10^7^ CFU/g	Oral with feed	Higher immune-related gene expression after being challenged with *V. alginolyticus* and *V. parahaemolyticus*	60 days	10	[68]
12.	*B. subtilis*, lactic acid bactria	10^5^ CFU (g diet) of *B. subtilis* and 10^5^ CFU (g diet) of LAB, 10^6^ CFU (g diet) of *B. subtilis* and 10^6^ CFU (g diet) of 1 LAB	Oral with feed	Reduce *Vibrio* spp. infection by improving immunity and intestinal microbiota	12 weeks	200	[48]
13.	*Bacillus coagulans*	1 × 10^6^ (BC1), 1 × 10^7^ (BC2), and 1 × 10^8^ (BC3) CFU g 1 of feed	Oral with feed	Increase the resistance against *V. parahaemolyticus* infection (especially 1 × 10^8^ CFU showed 76% survival rate after infection)	56 days	40	[44]
14.	*Monascus purpureus* M-32	5 g/kg, 10 g/kg, and 20 g/kg	Oral with feed	Higher immune-related gene expression after a challenge by *V. parahaemolyticus*	8 weeks	40	[69]
15.	*Lactobacillus pentosus* HC-2	5 × 10^8^ CFUg/feed	Oral with feed	Showed the expression levels of immune genes and 49% of survival rate with the infection of Aflatoxin B1	6 weeks	100	[70]
16.	*P. polymyxa* ATCC 842	10^6^ (PP1), 10^7^ (PP2) and 10^8^ (PP3) CFU/g of P. polymyx	Oral with feed	Reduce the abundance of opportunistic bacterial pathogens (*Vibrio*, *Photobacterium, Tenacibaculum,* and *Shewanella*), especially 10^8^ showed 78.3% survival rate after *V. parahaemolyticus*	8 weeks	40	[58]

CFU=Colony-forming units, LAB=Lactic acid bacteria, *P. polymyx=Paenibacillus polymyxa, V. parahaemolyticus=Vibrio parahaemolyticus*, *V. alginolyticus*=Vibrio alginolyticus

*Lactobacillus* and *Bacillus* spp. are groups of bacteria that are widely used as non-specific immune stimulants in vannamei shrimp, such as *Lactobacillus plantarum, Lactobacillus rhamnosus, Lactobacillus fermentum, Lactobacillus paracasei, Lactobacillus pentosus, Bacillus subtilis, Bacillus licheniformis, Bacillus pumilus*, and *Bacillus coagulans* [40, 71–74]. Other groups of bacteria are used as immune response or body resistance stimulants in vanamei shrimp, including *Pseudomonas*, *Nitrosomanas*, *Aerobacter*, and *Nitrobacter* spp., as well as *Rhodobacter sphaeroides*, *Clostridium butyricum*, and *Enterococcis faecium* [17, 52, 65, 75]. The provision of these bacteria increases lysosome production in shrimp. Lysosomes hydrolyze and break glycoside bonds in bacterial cell walls, thus rendering it more difficult for pathogenic bacteria to infect shrimp. Furthermore, lysosomes increase aspartate aminotransferase and alanine aminotransferase, which are indicators of natural immunity in shrimp, and also increase other defense cells in shrimp [46, 76]. If the body’s resistance to disease increases, then the occurrence of infections can be minimized and shrimp growth maximized.

Probiotics are well known for their antagonism to pathogens within the host species and in culture systems. Several mechanisms for this action are defined by the type of probiotic bacteria that induce bacterial antagonism, such as suppressing populations of *Vibrio* spp. [75]. The use of probiotics can further induce a competitive exclusion process that prevents pathogen infection by developing vital resistance genes [77]. For example, many *Bacillus* spp. can produce opportunistic antibiotics and metabolites in response to pathogenic microbes. For example, *Lactobacillus plantarum* Ep-M17 has been shown to increase digestive enzymes (trypsin) and antioxidant enzymes, increase shrimp immunity (76.9%), and increase survival rate (89%) [46]. These bacteria compete for space with other pathogenic bacteria, such as *Staphylococcus, Aerococcus*, and *Vibrio* spp., and *Escherichia coli* [40]. The previous studies found that AHPND-infected vannamei shrimp had increased amounts of *V. parahaemolyticus* in the gut [78]. The use of beneficial bacteria reduces the number of pathogenic bacteria in the gut. In addition, probiotics are a cheap, non-pathogenic, and largely non-toxic source of antibiotics that synthesize various metabolites with antibacterial functions, thus making them beneficial for commercial production [79]. Probiotics have also been used experimentally to control microbial pathogenicity in fish [80, 81].

## Effects on Water Quality

The addition of probiotics in vannamei shrimp aquaculture can affect water quality. Table-3 shows the role that probiotics play in maintaining good water quality [12, 82–90]. The addition of probiotics or external bacteria, such as nitrifying bacteria or *Lactobacillus* and *Bacillus* spp., have been shown to affect dissolved oxygen, pH, ammonia, and alkalinity concentrations in water [12, 82, 83], since these bacteria oxidize ammonia to nitrite and convert nitrite to nitrate [91]. Ammonia in shrimp cultures can come from uneaten feed, feces, dead/decaying plankton, and airborne debris [92]. Ammonia is toxic and lowers the immune system and can even cause mortality [93]. Probiotic bacteria found in water also compete for space with pathogenic bacteria [94].

**Table-3:** The role of probiotics in maintaining good water quality in vannamei shrimp culture.

S. No.	Probiotic	Dosage	Administration	Effect	Duration	Density	Reference
1.	*Lactobacillus rhamnosus*, Nitrification bacteria/*Nitrosococcus, Nitrococcus*	7.5 × 10^6^ and 10.6 × 10^3^ CFU/mL	Immersed in water (nitrification bacteria) and oral (*Lactobacillus rhamnosus*)	Good water quality, especially adding water probiotics showed less TAN, NO_2_-N	35 days	60	[82]
2.	*Bacillus infantis* and *Bacillus* (commercial probiotic)	1 × 10^9^ CFU/mL	Immersed in water	Good values of water quality	110 days	400,000	[85]
3.	*Bacillus* spp.	1×10^5^ CFU/mL	Oral with feed	Reduce total ammonia and maintain good water quality	5 weeks	25	[12]
4.	*Bacillus* spp. Probiotic A (*Bacillus* thuringiensis BUU 001, *B. megaterium* BUU 002, *B. polymyxa* BUU 003, B. licheniformis BUU 004, *B. subtilis* BUU 005) and Probiotic B (*B. subtilis* BUU 006, *B. polymyxa* BUU 007, *B. megaterium* BUU 009, *B. circulans* BUU 010, *B. pumilus* BUU 012.).	10^10^ CFU/mL in the form of freeze-dried, microencapsulated, artemia enrichment	Oral as natural feed enrichment	Enhanced water quality, especially pH, ammonia, nitrite	22 days	1000 zoea and 500 postlarvae (shrimp)	[83]
5.	*Bacillus* coagulans SC8168	1 × 10^6^ CFU/mL 1 × 10^5^ CFU/mL 5 × 10^5^ CFU/mL	Immersed in water	Good water quality	Until post-larvae 7–8	100	[88]
6.	Probiotic A (viable *Bacillus*), Probiotic B (multi spp.)	5 × 10^10^ spores/g (Probiotic A) 2 × 10^9^ CFU/g (Probiotic B)	Immersed in water	Good water quality (both reducing TVC and NH_3_ by increasing dissolved oxygen and pH in the pond water)	8 weeks	250,000	[87]
7.	*L. plantarum*, *Lactobacillus* *fermentum*, *B. subtilis*, B. licheniformis, *B. megaterium*, *Nitrobacter* spp., and *Nitrosomonas* spp.	1 mL/10 L (P1), 2 mL/10 L (P2), 3 mL/10 L (P3), 4 mL/10 L (P4)	Immersed in water	Good water quality	9 months	5	[89]
8.	Lactic acid bacteria (*Lactobacillus reuteri, Pediococcus acidilactici*)	10^3^, 10^5^, and 10^7^ CFU/g	Oral with feed	Lowest ammonia-nitrogen concentration at the *Pediococcus acidilactiti*	8 weeks	150	[86]
9.	*B. licheniformis*	2 mg/L and 4 mg/L	Oral with feed	Decrease NH_4_^+^-N, NO_2_^−^-N, and NO_3_^−^-N	20 days	20,000	[90]
10.	Probiotic 1 (*B. amyloliquefaciens* Ba-BPD1), Probiotic 2 (*B. amyloliquefaciens* F001)	50 mL	Immersed in water	Increase nitrite-nitrogen and nitrate-nitrogen	40 days	150	[84]

*B. megaterium*=*Bacillus megaterium, B. polymyxa*=*Bacillus polymyxa, B. licheniformis*=*Bacillus licheniformis,* *B. subtilis*=*Bacillus subtilis, B. circulans*=*Bacillus circulans, B. pumilus*=*Bacillus pumilus, L. plantarum*=*Lactobacillus plantarum, B. amyloliquefaciens*=*Bacillus amyloliquefaciens*, CFU=Colony-forming units

Surprisingly, the use of probiotics in water reduces the dissolved oxygen content in the water, even though it is still within the safe limits for the needs of vannamei shrimp (>5 mg/L) [84, 95]. This could be a consequence of the greater abundance of bacteria in the water when probiotics are administered to it. The increased number of bacteria in the water increases the competition for oxygen [96]; therefore, the proper probiotic dose needs to be administered and monitored to avoid a detrimental decrease in the amount of dissolved oxygen present in the culture.

Probiotics are also used in biofloc systems for vannamei shrimp production [82, 85] and have been shown to maintain good water quality in these systems as well. The probiotics promote water quality through nitrification and denitrification and induce biofloc formation [97]. Biofloc is a collection of various organisms (e.g., bacteria, fungi, algae, protozoa, and worms) and organic matter that are incorporated in a floc. The shrimp eat the floc so that no organic matter is harmful to shrimp [98]. Floc eaten by shrimp can reduce feed use and FCR.

The administration of probiotics to food and, thus indirectly to water has also been shown to improve water quality. For example, the provision of dietary *Pediococcus acidilactiti* reduces the ammonia–nitrogen concentration in the water [86]. This is a unique finding since the previous research typically has only shown the positive effect of probiotics on water quality with their direct addition to water [87, 88].

## Conclusion and Future Outlook

Over the past few decades, pathogenic bacteria and viruses have become the dominant cause of shrimp aquaculture diseases. Antibiotics, which are expected to counter these diseases, are often faced with backlash as they also endanger the host organism and the consumer [60, 99]. The inappropriate use of antibiotics, which is sometimes widely practiced, causes the bacteria to become resistant to antibiotics. Thus, the search for a more friendly alternative has been increasing, with probiotics becoming a promising candidate. As dietary supplements, probiotics enhance the competitive exclusion of pathogens from the aquaculture system and improve the shrimp’s immune parameters without affecting its health. Probiotics can be considered a better alternative to antibiotics and similar products to protect and maintain environmental stability. Further experiments have confirmed that probiotic supplementation in shrimp feed can significantly reduce disease occurrence and improve the enzymatic activities of feed consumption, growth, and shrimp survival [100].

Despite these benefits, there are concerns when probiotics are applied in inappropriate amounts, which may lead to excessive nutrient production and microbial disturbances. In conclusion, in-depth knowledge of the genetic makeup and transcriptomic and proteomic profiles of probiotics is greatly needed to improve methodical and comprehensive field applications of probiotic use in shrimp farming [101]. Other natural alternatives, namely, paraprobiotics (non-biological counterparts of probiotic organisms), algae, and plant extracts containing prebiotic properties also need further development[102–105]. Natural products can provide comparable beneficial effects and reduce production costs. Finally, this review reported that probiotics have higher effectiveness in increasing shrimp yield compared to antibiotics. However, this finding still requires additional research into the molecular pathways that regulate the probiotic mechanisms that affect shrimp metabolism.

## Authors’ Contributions

MKA: Conducted the study and drafted the manuscript. AFL, RBP, MR, and SMEP: Designed the study and drafted the manuscript under the guidance of MBS. MAAS and MTJ: Data analysis and revised the manuscript. All authors have read, reviewed, and approved the final manuscript.
